# Troponin I as a prognostic marker for outcomes in septic patients admitted to the ICU: Evidence from a government medical college of Madhya Pradesh, India

**DOI:** 10.6026/973206300223125

**Published:** 2026-05-31

**Authors:** Vikas Rangare, Jyoti Nagwanshi1, Abhay Kumar, Kapil Raghuwanshi

**Affiliations:** 1 Department of Medicine, Chhindwara Institute of Medical Sciences, Chhindwara, Madhya Pradesh, India; 2 Department of ENT, Chhindwara Institute of Medical Sciences, Chhindwara, Madhya Pradesh, India; 3 Department of Biochemistry, Chhindwara Institute of Medical Sciences, Chhindwara, Madhya Pradesh, India

**Keywords:** Sepsis, Troponin I, ICU mortality, septic cardiomyopathy, sequential organ failure assessment (SOFA) score, biomarkers, critical care

## Abstract

Sepsis remains a leading cause of morbidity and mortality among critically ill patients, with early identification of high-risk individuals being vital for improving outcomes. Cardiac troponin I, a biomarker of myocardial injury, is often elevated in sepsis and may serve as an indicator of systemic inflammation and cardiovascular stress. Hence, this prospective observational study conducted from December 2024 to January 2026 at District Hospital Chhindwara, India, included 170 adult sepsis patients admitted to the ICU. Troponin I levels within 24 hours of admission were measured and analyzed against clinical variables including SOFA score; mechanical ventilation, vasopressor use and length of ICU stay. Elevated troponin was detected in 67.6% of patients and was significantly associated with higher mortality (38.3% vs. 9.1%, p < 0.001) and greater need for organ support; logistic regression confirmed troponin as an independent mortality predictor (adjusted OR = 5.12, 95% CI 1.65-15.9, p = 0.004). Thus, we show troponin I as a valuable prognostic marker in sepsis, supporting its early measurement for risk stratification and targeted management.

## Background:

Sepsis is a life-threatening syndrome caused by a dysregulated host response to infection, resulting in organ dysfunction [1]. It continues to be one of the main causes of morbidity and mortality among patients in ICUs and a significant global health burden. Despite advances in antimicrobial therapy, early recognition and supportive care, sepsis continues to carry a high mortality. The clinical course is unpredictable-some patients recover quickly, while others develop rapid multiorgan failure. Early identification of high-risk patients is crucial to enable timely interventions that may improve outcomes. To assess organ dysfunction and predict results, clinical scoring methods like the Sequential Organ Failure Assessment (SOFA) score are often employed [2]. Six organ systems are assessed by the SOFA score: respiratory, cardiovascular, renal, hepatic, neurological and coagulation. A high score is related to a high mortality rate. Although useful, such scoring systems only identify variations that arise when an organ is damaged and therefore, it is desirable to have early biochemical markers that will give extra prognostic information [3]. The pathophysiology of sepsis involves complex interactions between pathogens and the host immune response. Infection triggers inflammatory pathways, releasing cytokines and other mediators. Uncontrolled inflammation may lead to oxidative stress, tissue damage and dysfunction of the endothelium, disturbed vascular permeability, dysfunctional microcirculation and metabolic imbalances, which may lower the tissue oxygen supply and lead to organ dysfunction. As a result, there is a high likelihood of simultaneous multiple organ system involvement [4]. Cardiac biomarkers, particularly troponins, have emerged as useful indicators of myocardial involvement in critically ill patients [4]. When cardiac cells are injured, the very sensitive and specific indicators of myocardial injury, troponin I and troponin T, are released into the bloodstream. It has been proven clinically that high levels of troponin in septic patients correlate with the severity of the disease and a greater number of patients dying [5]. The meta-analyses have strengthened the prognostic value of troponin elevation in sepsis [6, 7]. Even a small increase in troponin in the critically ill patients without underlying cardiac disease is associated with worse outcomes, indicating that troponin is a marker of systemic physiological stress and dysfunction of the myocardium in severe infection [8]. Myocardial dysfunction associated with sepsis is characterized by decreased ventricular contractility, decreased cardiac output and lack of response to circulatory demands, which occur even in subjects with no underlying cardiac diseases [9]. Therefore, it is of interest to determine whether ICU mortality and Troponin I levels at the time of ICU admission were related and examine the relationship between the need for organ support in critically ill patients experiencing septic shock, the severity of illness and Troponin I elevation.

## Materials and Methods:

This prospective observational study had been carried out at District Hospital Chhindwara, associated with Chhindwara Institute of Medical Sciences, Madhya Pradesh, India, from December 2024 to January 2026. Approval had been obtained from the Institutional Human Ethics Committee (approval number: CIMS/Ethics Committee/2024/14611).

## Study population:

Adult patients admitted to the ICU with sepsis, defined based on Sepsis-3 criteria, were consecutively enrolled and followed until discharge or death. Patients who had acute myocardial infarction, severe heart failure, severe valvular disease, end-stage renal disease under dialysis and acute coronary syndrome were excluded to ensure that troponin elevations were caused by sepsis [10, 11].

## Sample size:

Sample size calculations were based on prior studies linking troponin elevation to mortality in sepsis [12]. A total of 170 patients were enrolled prospectively to achieve adequate statistical power.

## Data collection:

Standardized forms were used to record clinical and laboratory data. Demographics, source of infection, SOFA score at admission, mechanical ventilation, and the use of vasopressors and the length of stay in the ICU were gathered. Measured serum Troponin I within 24 hours of admission to the ICU, with the levels being classified as high or normal. Recording of mechanical ventilation requirements and vasopressor support requirements was done, whether initiated at ICU admission or at any point in the ICU stay. Markers of organ dysfunction were mechanical ventilation and vasopressor support.

## Outcome measures:

The primary outcome was ICU mortality. Troponin I status was the primary exposure variable. Other variables such as age, gender, SOFA score, mechanical ventilation, vasopressor support and source of infection were also evaluated as possible predictors. Length of stay at the ICU was recorded but not fitted into the regression models so as to prevent reverse causality.

## Statistical analysis:

Data were examined utilizing conventional descriptive and inferential statistical techniques. Continuous data were evaluated for normality and presented as mean ± standard deviation (SD) or median with interquartile range (IQR), as applicable, while categorical variables were summarized as frequencies and percentages. A chi-square test had been employed to compare groups in terms of categorical variables, whereas the Mann-Whitney U test was utilized to compare groups in terms of continuous data. When expected cell counts were small (n < 5), the Fisher exact test was applied to assess differences in categorical outcomes, as in the comparison of ICU mortality between patients with elevated versus normal Troponin I levels in the lowest SOFA category (<8). The differences among different groups had been tested using the Kruskal-Wallis test. A multivariate logistic regression analysis identified the independent determinants of ICU mortality, which were given as adjusted ORs (Odds Ratios) and CIs (confidence intervals). The goodness-of-fit test based on Hosmer-Lemeshow and the area under the receiver operating characteristic curve (AUC) was employed to determine the performance of the model. The p-value of 0.05 had been considered statistically significant on both sides. SPSS version 26.0 had been employed to conduct statistical tests.

## Data management:

Two independent investigators collected data, resolving discrepancies by consensus. Missing data (<2%) were addressed with complete-case analysis. To ensure confidentiality, patient identifiers were removed. This method ensured reproducible and good-quality data.

## Results:

The analysis comprised 170 adult sepsis patients who had been admitted to the ICU. The group's average age was 58.7 ± 11.6 years. There were 93 males (54.7%) and 77 females (45.3%). Elevated Troponin I levels were observed in 115 patients (67.6%), whereas 55 patients (32.4%) had values within the normal range. Overall ICU mortality was 29.0% (49/170), while 121 patients (71.0%) survived to ICU discharge. The total group's median ICU stay was 7 days (IQR: 4-10) (Table 1). Patients with raised Troponin I levels experienced a notably prolonged stay in the ICU compared with those whose levels were within the normal range. The median duration of stay was 9 days (IQR 5-12) in the elevated group, whereas it was 4 days (IQR 3-6) among patients with normal values. This difference was found to be statistically significant based on the Mann-Whitney U test (U = 895, p < 0.001). Further analysis based on the source of infection revealed a significant variation in ICU length of stay across different groups (Kruskal-Wallis test: H (2) = 35.2, p < 0.001). Among these, patients diagnosed with pneumonia tended to have the longest ICU stays, followed by those with abdominal infections, while individuals with urinary tract infections generally had shorter stays (Table 2). ICU mortality differed markedly according to Troponin I status. Among patients with elevated troponin levels, 44 of 115 (38.3%) died during ICU admission, compared with 5 of 55 patients (9.1%) in the normal troponin group. Survivors included 71 patients in the elevated troponin group and 50 patients in the normal group. The association between troponin status and mortality was statistically significant on Chi-square analysis (χ^2^ = 21.7, DF = 1, p<0.001) (Table 3). Patients with increased Troponin I levels showed a markedly higher need for organ support during their ICU stay. A substantially greater proportion of these patients required mechanical ventilation compared to those with normal Troponin levels (78.3% vs. 25.5%) and this difference was statistically significant (χ^2^ (1) = 53.6, p p< 0.001). In the same way, the use of vasopressors was considerably more common among patients with elevated Troponin I, with 83.5%, requiring support versus 21.8% in the normal group. This association was also statistically significant (χ^2^ (1) = 68.2, p < 0.001) (Table 4). Multivariable logistic regression identified several independent predictors of ICU mortality. Increasing age was associated with higher mortality (OR 1.04 per year; 95% CI 1.01-1.08; p = 0.022). The SOFA score was a strong predictor, with each point increasing the odds of death by 52% (OR 1.52; 95% CI 1.28-1.82; p < 0.001). The need for mechanical ventilation (OR 2.31; p = 0.033) and vasopressor support (OR 2.84; p = 0.008) were also independently associated with mortality. Elevated Troponin I remained a significant predictor (OR 5.12; p = 0.004), whereas male gender was not (p = 0.56) (Table 5). The model showed good calibration (Hosmer-Lemeshow χ^2^ (8) = 6.12, p = 0.63) and excellent discrimination (AUC = 0.86). Mortality increased with rising SOFA scores and was consistently higher in patients with elevated Troponin I. In the low SOFA group (<8), mortality was 15% for patients with elevated Troponin compared to 4% for those with normal levels, though this difference was not statistically significant (Fisher's exact test, p = 0.09). In the intermediate SOFA category (8-10), mortality was significantly greater in the elevated Troponin group (33% vs. 10%; χ^2^ (1) = 5.3, p = 0.02). Similarly, among patients with SOFA scores >10, those with elevated Troponin I had higher mortality than those with normal levels (52% vs. 20%; χ^2^ (1) = 4.8, p = 0.028) (Table 6). In this study, elevated Troponin I at ICU admission was independently related to higher mortality among septic patients. Patients with high troponin not only stayed longer in the ICU but also needed more mechanical ventilation and vasopressors. Notably, Troponin I further stratified prognosis in all categories of SOFA scores and it is important to note that myocardial injury is a major determinant of the severity and outcome of sepsis. The findings provide support for the use of Troponin I as a biomarker for prognostication and early risk assessment in critically ill sepsis patients.

## Discussion:

This prospective study assessed the predictive significance of Troponin I levels measured upon ICU admission in patients with sepsis. Adverse outcomes such as high mortality rates, extended ICU stay and increased mechanical ventilation and vasopressor dependence had a significant correlation with elevated levels of Troponin I. However, it is important to note that the relationship remained significant when age and severity of illnesses were included in the SOFA score and this implies that Troponin I is more prognostic than the conventional clinical variables. Cardiac involvement is increasingly recognized as an important component of sepsis. Past studies have established that troponin increase in the case of septic patients can be an indicator of myocardial infarction and can be associated with the disrupted ventricular activity, known as septic cardiomyopathy [13, 14]. In contrast to acute coronary syndromes, where myocardial injury is caused by coronary occlusion, troponin release during sepsis is considered to be caused by systemic inflammatory reactions, microvascular dysfunction and metabolic changes of cardiomyocytes [15]. The pathways that cause myocardial injury in sepsis are complex and comprise nitric oxide imbalance, dysfunction of mitochondria and disordered myocardial energy metabolism that can result in myocardial depression and release of troponin [16]. Therefore, high levels of Troponin I in septic patients could be the manifestation of myocardial stress caused by systemic diseases, not the primary ischemic heart disease. Scoring systems like SOFA and APACHE II are commonly used to assess the severity of a disease and they mainly mirror established organ dysfunction. Troponin I measurement can thus be used as a supplementary biomarker that can give extra information about cardiovascular involvement. Non-cardiac ICU population studies have indicated that higher troponin levels are connected to a higher mortality rate and unfavorable clinical results, which indicates that troponin could be a more generic predictor of overall disease [17]. The findings of the current study correspond with these results and support the use of Troponin I as an effective marker for risk classification in septic patients. Advances in laboratory diagnostics have further improved the detection of myocardial injury. High-sensitivity troponin assays allow identification of minimal cardiomyocyte damage that may otherwise remain undetected, facilitating earlier recognition of septic cardiomyopathy [18]. Additionally, some studies have reported that persistently rising troponin levels during septic shock are associated with greater hemodynamic instability and poorer short-term outcomes, indicating that serial measurements may provide further prognostic information [19]. Imaging studies have also contributed to the understanding of cardiac dysfunction in sepsis. Echocardiographic evidence suggests that myocardial impairment in septic patients is often reversible once the underlying infection is controlled. Combining cardiac biomarkers with established severity scores has therefore been proposed as a strategy to improve prognostic assessment in critically ill patients [20]. Overall, the results of this study and existing literature demonstrate that troponin elevation in sepsis reflects clinically meaningful myocardial injury within the context of systemic inflammation and multiorgan dysfunction. The assessment of Troponin I upon ICU admission could assist clinicians in identifying patients with an elevated risk of negative outcomes in the intensive care setting. This prospective study aimed to examine the prognostic usefulness of Troponin I in ICU patients with sepsis. The elevated troponin levels were closely associated with increasing mortality rates, extended ICU lengths of stay and the need to support organs. Remarkably, even with the age and severity of illness assessed, Troponin I remained an independent predictor of ICU mortality and this shows that the myocardial damage provides better predictive data than traditional scoring systems. Numerous limitations must be considered. The research was conducted in one centre, which might not be applicable to the other settings. The Troponin I level was only assessed in the initial 24h of ICU admission and no serial measurements were done to determine dynamic changes with time. Also, full echocardiographic data were not always available, which restricted a direct assessment of myocardial dysfunction. Troponin I during ICU admission can be used to assess the potential for better outcomes in relation to septic patients. The elevated levels are most likely to be evidence of myocardial stress and a more serious systemic disease. Such patients may be observed more closely and timely supportive measures can be provided in case of their timely identification. Bringing the assessment of troponin into the routine examination of patients with septic conditions can improve the practice of critical care. Future studies should examine the value of serial Troponin I measurements to better understand the evolution of myocardial injury during sepsis. These findings also necessitate bigger, multicenter research to verify them and clarify whether troponin-directed management methods can improve clinical results. Integration of cardiac biomarkers and conventional severity scores can also enhance the subsequent phase of prognostic assessment and possibly personalised care of patients with septic shock.

## Conclusion:

We show the prognostic value of measuring troponin I at ICU admission in patients with sepsis, as elevated levels were closely linked to increased mortality, prolonged ICU stay and greater need for organ support. The findings suggest that myocardial injury represents a significant component of the systemic response to severe infection and that troponin I complements existing severity indices to refine patient risk assessment. Incorporating early troponin I screening into sepsis management may enhance clinical decision-making and enable more precise allocation of intensive care resources for high-risk patients.

## Figures and Tables

**Table 1:** Baseline characteristics of the study population

**Variable**	**Total (n = 170**
Age, years (mean ±SD)	58.7 ±11.6
Male, n (%)	93 (54.7)
Female, n (%)	77 (45.3)
Elevated Troponin I, n (%)	115 (67.6)
Normal Troponin I, n (%)	55 (32.4)
ICU mortality, n (%)	49 (29.0)
ICU survivors, n (%)	121 (71.0)
ICU length of stay, median (IQR)	7 (4-10)

**Table 2:** ICU Length of stay according to troponin I Status and infection source

**Variable**		**n**	**Median ICU Stay (days)**	**IQR**	**Statistical test**	**Test statistics**	**df**	**p-value**
Troponin I Status	Elevated	115	9	5-12	Mann-Whitney U	U=895	-	<0.001
	Normal	55	4	3-6				
	Pneumonia	60	9	7-12				<0.001
	Abdominal infection	50	7	5-9				
Infection Source	Urinary tract infection	60	4	3-5	Kruskal-Wallis H	H=35.2	2	

**Table 3:** ICU Mortality according to troponin I Status

**Troponin I Status**	**Total Patients**	**Deaths**	**Survivors**	**Mortality (%)**	**Statistical test**	**Test statistics**	**p-value**
Elevated	115	44	71	38.3	Chi-square	χ^2^ = 21.7, df = 1	<0.001
Normal	55	5	50	9.1			
Total	170	49	121	29			

**Table 4:** Organ support requirements according to troponin I Status

**Troponin I Status**	**Mechanical Ventilation n (%)**	**Vasopressor Support n (%)**	**Statistical test**	**Test statistics**	**df**	**p-value**
Elevated	90 (78.3)	96 (83.5)		χ^2^ = 53.6 (MV)	1	<0.001
Normal	14 (25.5)	12 (21.8)	Chi-square	χ^2^ = 68.2 (VP)	1	<0.001

**Table 5:** Multivariable logistic regression analysis for ICU Mortality

**Variable**	**Adjusted Odds Ratio**	**95% CI**	**p-value**	**df**
Age (per year)	1.04	1.01-1.08	0.022	1
Male gender	1.18	0.63-2.20	0.56	1
SOFA score (per point)	1.52	1.28-1.82	<0.001"	1
Mechanical ventilation	2.31	1.07-4.98	0.033	1
Vasopressor support	2.84	1.31-6.12	0.008	1
Elevated Troponin I	5.12	1.65-15.9	0.004	1

**Table 6:** ICU Mortality according to SOFA score category and troponin I status

**SOFA Category**	**Troponin I Elevated (n)**	**Deaths (%**	**Troponin I Normal (n)**	**Deaths (%)**	**Statistical Test**	**Test Statistic**	**df**	**p-value**
< 8	20	3 (15%)	25	1 (4%)	Fisher's Exact	-	-	0.09
8-10	45	15 (33%)	20	2 (10%)	Chi-square	χ^2^ = 5.3	1	0.02
>10	50	26 (52%)	10	2 (20%)	Chi-square	χ^2^ = 4.8	1	0.028
